# Supplementary material for: Electrochemical characterization of manganese oxides as a water oxidation catalyst in proton exchange membrane electrolysers
Source: R Soc Open Sci. 2019 May 22;6(5):190122. doi: 10.1098/rsos.190122 (PMC6549974; doi:10.1098/rsos.190122)
Supplement: Scanning electron microscope (SEM) images [file rsos190122supp1.docx]

Supplemental Material

Electrochemical Characterization of Manganese Oxides as a Water Oxidation Catalyst in PEM Electrolyzers

Toru Hayashi^1,2^, Nadège Bonnet-Mercier^2^, Akira Yamaguchi^2,†^, Kazumasa Suetsugu^3^, and Ryuhei Nakamura^2,4^*

^1^ Department of Applied Chemistry, The University of Tokyo, 7-3-1 Hongo, Bunkyo-ku, Tokyo 113-8656, Japan

^2^ Biofunctional Catalyst Research Team, RIKEN Center for Sustainable Resource Science (CSRS), 2-1 Hirosawa, Wako, Saitama 351-0198, Japan
E-mail: ryuhei.nakamura@riken.jp

^3^ Tosoh Corporation, 3-8-2 Shiba, Minato-ku, Tokyo 105-8623, Japan

^4^ Earth-Life Science Institute (ELSI), Tokyo Institute of Technology, 2-12-1, Ookayama, Meguro-ku, Tokyo 152-0033, Japan

^†^ Present address: Department of Materials Science and Engineering, School of Materials and Chemical Technology, Tokyo Institute of Technology, S7-9, 2-12-1 Ookayama, Meguro-ku, Tokyo 152-8552, Japan

**Figure S1.** Scanning electron microscope (SEM) images of (a) the carbon black (Vulcan XC-72) and (b) the mixture of the *γ*-MnO_2_ and the carbon black (Scale bar: 200 nm).
